# Capturing macromolecular dynamics using time-resolved serial crystallography at X- ray free electron lasers and synchrotron light sources

**DOI:** 10.1063/4.0000957

**Published:** 2025-10-27

**Authors:** Sandra Mous, Guillaume Gotthard, David Ehrenberg, Saumik Sen, Tobias Weinert, Philip Johnson, Daniel James, Karol Nass, Antonia Furrer, Pikyee Ma, Steffen Bruenle, Cecilia Casadei, Isabelle Martiel, Florian Dworkowski, Dardan Gashi, Petr Skopintsev, Maximilian Wranik, Gregor Knopp, Ezequiel Panepucci, Valerie Panneels, Claudio Cirelli, Dmitry Ozerov, Gebhard Schertler, Meitian Wang, Chris Milne, Joerg Standfuss, Igor Schapiro, Joachim Heberle, Przemyslaw Nogly

**Affiliations:** 1 Linac Coherent Light Source, SLAC National Accelerator Laboratory, Menlo Park, CA, USA; 2 Paul Scherrer Institute, Villigen, AG, Switzerland; 3 Freie Universität Berlin, Berlin, BE, Germany; 4 University of California, Berkeley, CA, USA; 5 Stanford University, Palo Alto, CA, USA; 6 Hebrew University of Jerusalem, Jerusalem, JM, Israel; 7 Jagiellonian University, Krakow, MA, Poland

## Abstract

In recent years, time-resolved serial crystallography has emerged as a transformative technique for unraveling the intricate dynamics of macromolecules at atomic resolution. By leveraging the high-intensity and ultra-short pulses of X-ray free electron lasers (XFELs) alongside the high brilliance of synchrotron light sources, this technique has enabled the observation of transient states in biomolecules as they catalyze chemical reactions.

This presentation will highlight the advancements and applications of time-resolved serial crystallography in the study of macromolecular dynamic. We will discuss the light-sensitive membrane protein *Nonlabens marinus* halorhodopsin (*Nm*HR) as an example of how this method enables us to capture the structural dynamics from femtoseconds to milliseconds after light activation. Through combining time-resolved studies at the X-ray free electron laser and synchrotron with spectroscopy and chemical simulation, we obtained a comprehensive understanding of the molecular mechanism that allows *Nm*HR to catalyze ion transport across biological membranes. In addition to discussing the rich chemical information that can be obtained in time-resolved crystallographic studies, this talk will highlight how steady-state experiments can provide exciting structural insights while requiring only a limited amount of beamtime and a minimal setup.

